# Supplementary material for: DMRforPairs: identifying Differentially Methylated Regions between unique samples using array based methylation profiles
Source: BMC Bioinformatics. 2014 May 15;15:141. doi: 10.1186/1471-2105-15-141 (PMC4046028; doi:10.1186/1471-2105-15-141)
Supplement: Additional file 2 — DMRforPairs output for the comparison of A431-MCF7 and NA17018-NA17105. Please start from the HTML files in each folder. Available via the BMC Bioinformatics website. [file 1471-2105-15-141-S2.zip › 1394847754114233_MOESM2_ESM/A431_MCF7/figures/14289.pdf]

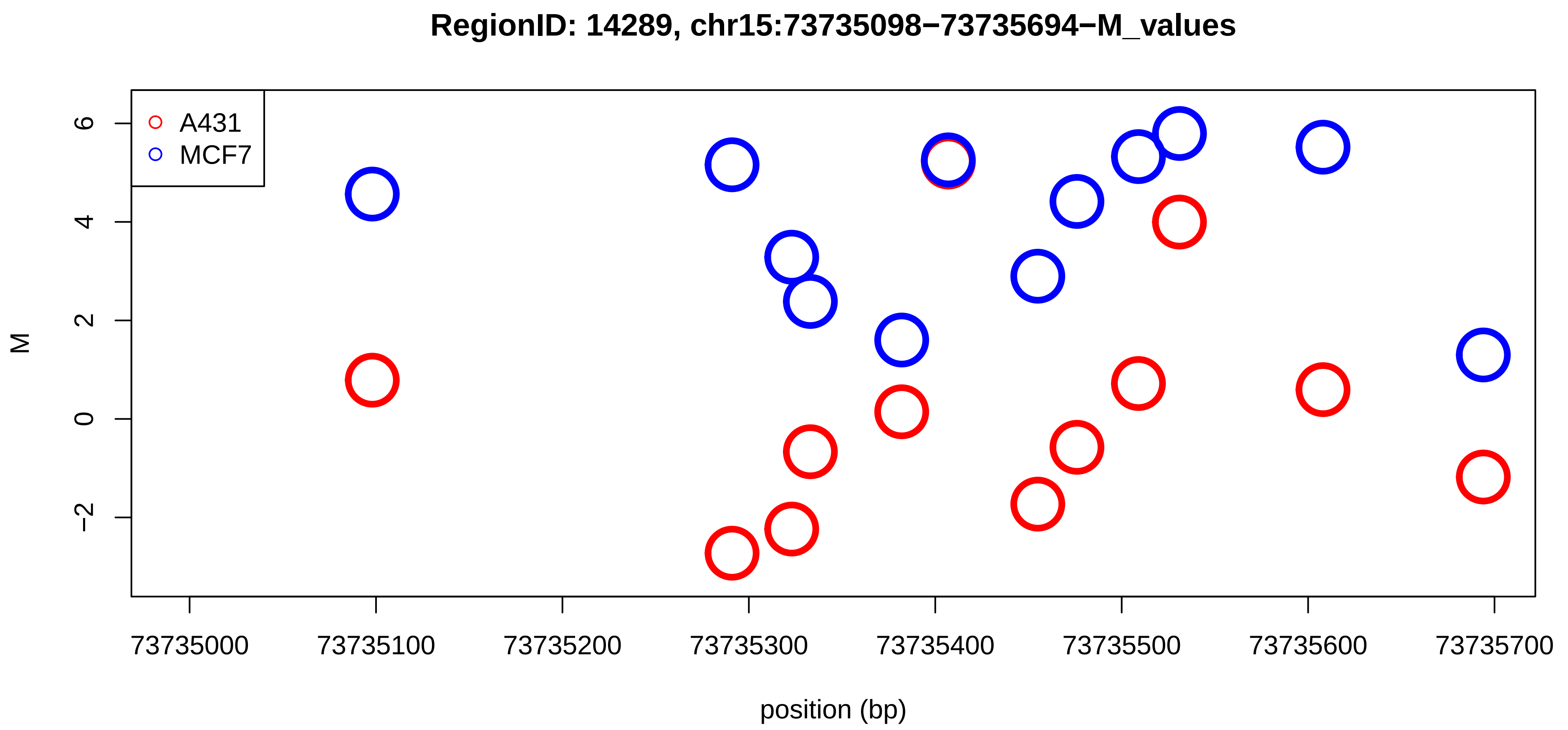

RegionID: 14289, chr15:73735098−73735694−M_values
A431
MCF7
M
6
4
2
0
−2
73735000
73735100
73735200
73735300
73735400
73735500
73735600
73735700
position (bp)

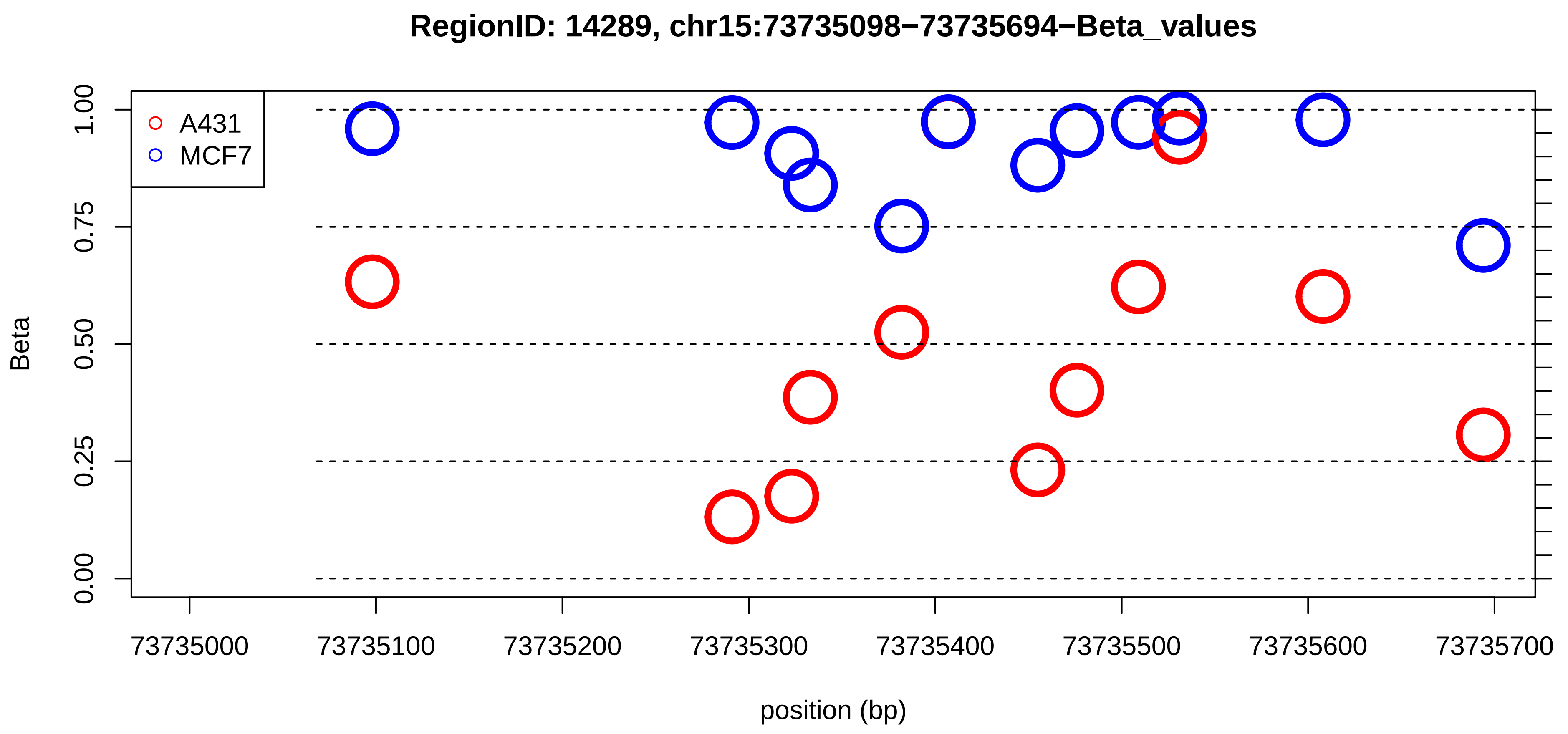

RegionID: 14289, chr15:73735098−73735694−Beta_values
A431
MCF7
Beta
1.00
0.75
0.50
0.25
0.00
73735000
73735100
73735200
73735300
73735400
73735500
73735600
73735700
position (bp)

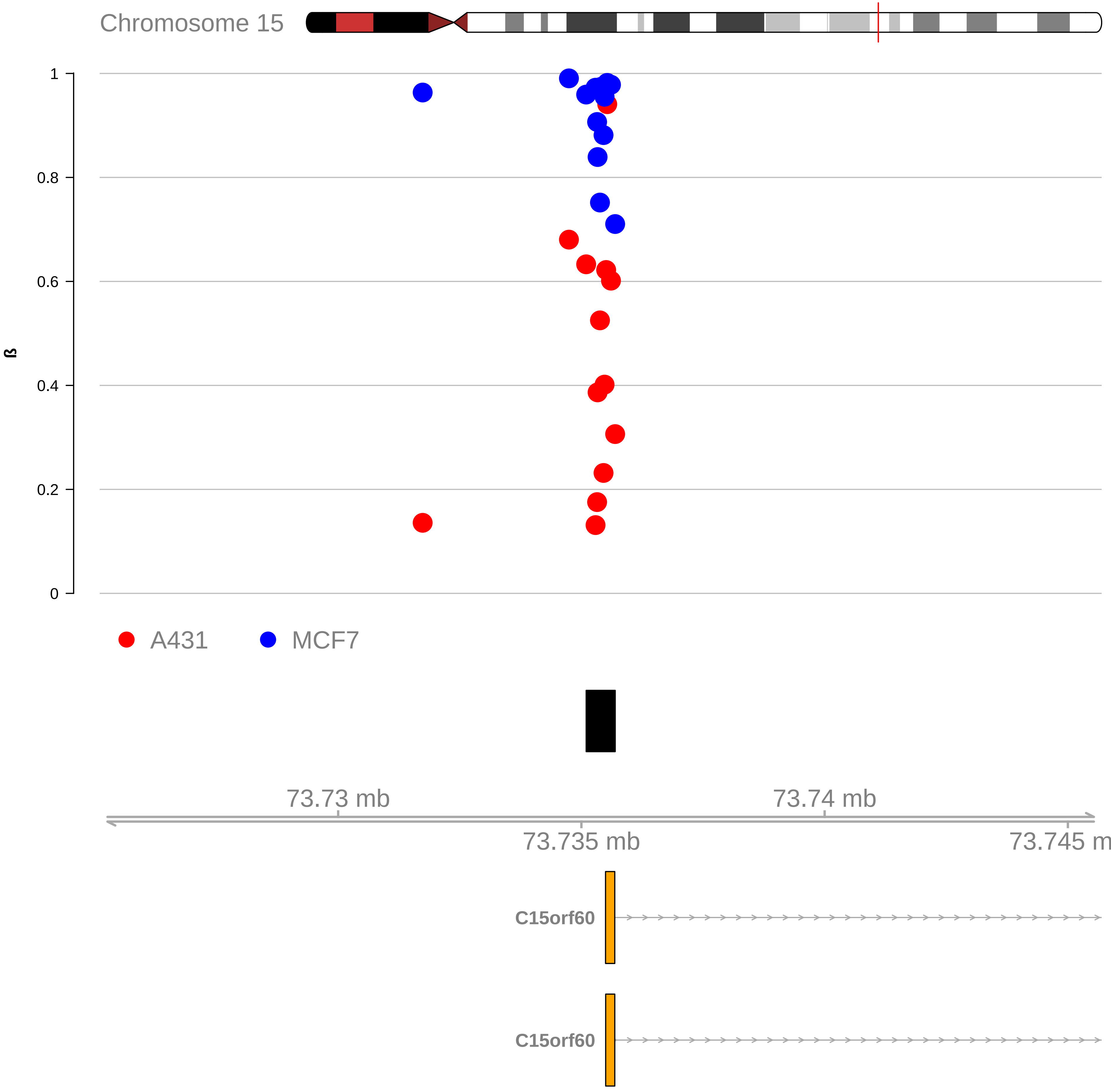

Chromosome 15
1
0.8
0.6
0.4
0.2
0
ß
A431
MCF7
73.73 mb
73.74 mb
73.735 mb
73.745 m
C15orf60
C15orf60
